# Venezuelan Equine Encephalitis Virus, Southern Mexico

**DOI:** 10.3201/eid1012.040393

**Published:** 2004-12

**Authors:** José G. Estrada-Franco, Roberto Navarro-Lopez, Jerome E. Freier, Dionicio Cordova, Tamara Clements, Abelardo Moncayo, Wenli Kang, Carlos Gomez-Hernandez, Gabriela Rodriguez-Dominguez, George V. Ludwig, Scott C. Weaver

**Affiliations:** * University of Texas Medical Branch, Galveston, Texas, USA;; † Comision Mexico-Estados Unidos para la Prevencion de la Fiebre Aftosa y Otras Enfermedades Exoticas de los Animales, Mexico, Mexico City, Mexico;; ‡ U.S. Department of Agriculture, Fort Collins, Colorado, USA;; § Instituto Nacional de Investigaciones Forestales Agricolas y Pecuarias (INIFAP) Mexico City, Mexico;; ¶ U.S. Army Medical Research Institute of Infectious Diseases, Ft. Detrick, Maryland, USA;; # Instituto de Salud de la Secretaria de Salud de Chiapas, Tuxtla Gutierrez, Chiapas, Mexico

**Keywords:** encephalitis virus, Venezuelan equine encephalitis, arboviruses, alphavirus, seroprevalence, research

## Abstract

Equine epizootics of Venezuelan equine encephalitis (VEE) occurred in the southern Mexican states of Chiapas in 1993 and Oaxaca in 1996. To assess the impact of continuing circulation of VEE virus (VEEV) on human and animal populations, serologic and viral isolation studies were conducted in 2000 to 2001 in Chiapas State. Human serosurveys and risk analyses indicated that long-term endemic transmission of VEEV occurred among villages with seroprevalence levels of 18% to 75% and that medical personnel had a high risk for VEEV exposure. Seroprevalence in wild animals suggested cotton rats as possible reservoir hosts in the region. Virus isolations from sentinel animals and genetic characterizations of these strains indicated continuing circulation of a subtype IE genotype, which was isolated from equines during the recent VEE outbreaks. These data indicate long-term enzootic and endemic VEEV circulation in the region and continued risk for disease in equines and humans.

## Abstract 2

Evidence of enzootic and endemic Venezuelan equine encephalitis virus circulation in southern Mexico since the 1996 epizootic was obtained from serosurveys and virus isolations.

Venezuelan equine encephalitis (VEE) epidemics or epizootics involving hundreds of thousands of equine and human cases have occurred in the Americas since the 1930s (*1**,**2*). In Mexico, human VEE was first recognized during the 1960s along the Atlantic coast (*3**–**5*). In 1962, a total of 13 human cases were detected by serologic testing in Campeche and Champoton, state of Campeche (*3**,**6*); 5 deaths occurred (38% case-fatality rate), and 3 patients exhibited neurologic disease (*3*). A more extensive serosurvey in 1962–1964 in four southeastern states found 23 of 770 serum specimens had antibodies against VEE virus (VEEV; *Togaviridae*: *Alphavirus*) (*4*). Although clinical cases were not detected during that study, the findings implied extensive VEEV circulation. During 1963, VEEV subtype IE was recovered from a sentinel hamster and mosquitoes collected in southeastern Veracruz State (*7*). In 1965, a fatal human case occurred in the village of Jaltipan, Veracruz State (*5*). Almost simultaneously, equine epizootics were reported in 1966 in Tamaulipas State and in northern Veracruz State (*8*). Although no virus isolations were made, a VEEV etiology was suggested by serosurveys.

In 1969–1972, a major VEE outbreak began on the Guatemala-El Salvador border soon after an epizootic occurred in Ecuador and Peru. The Central American outbreak affected tens of thousands of equines and humans as it spread northward into Mexico. The first equine deaths were reported in 1969 in mountainous areas in La Trinitaria and La Concordia, Chiapas State, close to the Guatemalan border (*8*). By 1970, the epidemic and epizootic had caused 10,000 equine deaths and many unconfirmed human cases in the states of Chiapas and Oaxaca. By the end of 1972, nearly 50,000 equine deaths and 93 confirmed human deaths, as well as several hundred nonfatal human cases, had occurred in Mexico (*9**,**10*). The epidemic and epizootic eventually reached southern Texas, where ≈1,500 horses died and several hundred human cases occurred (*11*). This epizoodemic was caused by a subtype IAB strain and may have been halted in Texas by equine vaccination, insecticide spraying (*12*), and possibly by preexisting natural immunity to other alphaviruses in the equine population (*13*). Between 1973 and 1992, no VEE outbreaks were reported anywhere, prompting speculation that epizootic strains of VEEV had become extinct (*12*). However, reemerging VEEV activity in the early and mid-1990s in Venezuela and Colombia (*13**,**14*) and on the Pacific coast of southern Mexico (*15*) underscores the continued threat of VEE in the Americas.

Two equine epizootics occurred on the Pacific coast of Mexico in 1993 and 1996. In the summer of 1993 in coastal areas of Chiapas State, an outbreak affecting125 horses, with 63 deaths, was documented. Three years later, during the summer of 1996 in the adjacent State of Oaxaca, another equine epizootic involved 32 horses with 12 deaths (*15*). Both outbreaks were caused by a subtype IE VEEV strain. However, no human cases were documented during either outbreak. VEEV strains isolated from encephalitic horses during 1993 and 1996 produced little evidence of viremia in experimentally infected horses, although the strains had caused encephalitis (*16*). Viremia titers were similar to those generated by enzootic VEEV strains, which indicates that equines were probably not important amplifying hosts during either Mexican epizootic.

Two hypotheses could explain the sudden appearance of equine encephalitis on the Pacific coast of Mexico in 1993: 1) an equine-virulent VEEV variant was introduced into the region or 2) an enzootic variant circulating previously in the region became more virulent or began circulating at a higher level in 1993. To test these hypotheses, to determine whether VEEV has persisted since 1996, and to determine whether humans are affected by these viruses, we conducted surveillance during 2000–2001 in various coastal villages involved in the 1993 epizootic. Our results indicate that enzootic and endemic VEEV have been circulating in the region and that persons and horses face a continuing risk for disease.

## Methods

### Selection and Description of the Study Area

During the summer of 1999, the Mexican Agricultural Ministry was notified of a suspected VEE equine outbreak in coastal areas of Mapastepec and Pijijiapan Municipalities in the state of Chiapas. A total of 26 equine cases showing syndromes compatible with equine VEE were reported, with 23 deaths. All of the affected horses were 8–14 months of age with no VEEV vaccination history. Although necropsies were carried out on some equines, no viruses were isolated. However, three of the dead horses showed histopathologic changes suggestive of a nonrabies viral etiology, with VEEV hemagglutination inhibition (HI) antibody titers from 20 to 5,120 (R. Navarro-Lopez, unpub. data). Bovine serosurveys also indicated recent VEEV circulation in the area.

On the basis of these preliminary data, we selected several locations for further study within the La Encrucijada preserve, a coastal ecosystem of mangrove estuaries and mangrove forest located along the Pacific coastal plain in the southwestern portion of the State of Chiapas. The preserve is about 357,824 acres (1,440 km^2^) and is located from 14°43´ to 15°40´N and from 92°26´ to 93°20´W. The area is composed of coastal lagoons, swamps, and marshes forming the largest mangrove forest on the North American Pacific coast and is important for its biodiversity and flora, including the only zapotonal woodland (*Pachira acuatica*) of Mesoamerica. The flora include mangrove, zapote forest, marshes, evergreen seasonal forest, deciduous seasonal forest, coastal dune vegetation, and palm forest. The area supports a large variety of threatened wildlife, including 11 species of amphibians, 34 of reptiles, 294 of birds (94 migratory), and 73 of mammals (www.ramsar.org/profiles_mexico.htm).

**Figure 1:**
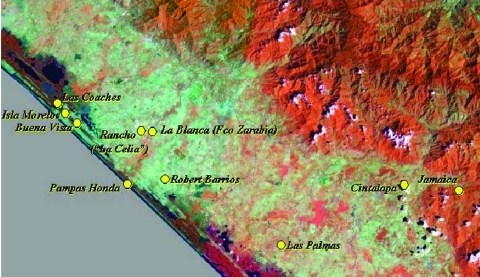
Satellite image of the Pacific coastal areas studied for Venezuelan equine encephalitis virus activity (Landsat thematic mapper). Bands 4, 5, and 1 are displayed as a red-green-blue false-color composite. The villages sampled are indicated in yellow.

About 30,000 persons live within 64 settlements in the preserve, and the main activities are commercial fishing, slash and burn agriculture, and extensive cattle ranching. We selected seven villages serologically implicated in VEEV circulation, all located between N 15°46´07´´–15°02´00´´ and W 93°4´92´´–92°43´00´´ and within the municipalities of Pijijiapan, Mapastepec, and Acapetahua (Figure 1). The villages included Las Coaches, Isla Morelos, Buenavista, 10 de Abril, Francisco Sarabia, Roberto Barrios, and Las Palmas (Table 1). Two other villages, Cintalapa and Jamaica, were selected as negative controls because they are well outside the area affected by the recent VEE epizootics in the foothills of the Sierra del Soconusco Mountains, 30–50 km from La Encrucijada. As part of ongoing dengue surveillance, serum samples from 434 persons from these different locales were tested for VEEV antibodies.

**Table 1:** Communities in the La Encrucijada Preserve studied for VEEV circulation and seroprevalence^a^

Community	Coordinates	Population size	Surveillance methods
Las Coaches	93°07´24´´ x 15°27´03´´	362	1,2,3
Isla Morelos	93°07´12´´ x 15°26´00´´	391	1
Buenavista	93°08´17´´ x 15°26´53´´	440	1
10 de Abril	93°02´04´´ x 15° 21´16´´	81	1
Francisco Sarabia	92°59´51´´ x 15°25´09´´	358	1
Roberto Barrios	92° 58´48´´ x 15°20´51´´	372	1,3
Las Palmas	92°45´14´´ x 15°34´51´´	832	1

^a^VEEV, Venezuelan equine encephalitis virus; 1, human serosurvey; 2, small mammal collections; 3, sentinel hamster exposure; data from Instituto Mexican del Seguro Social, Comision para las Areas Marginadas (rural clinics) as of July 2001.

### Animal Trapping and Collection of Serum Specimens

Recommended biosafety methods were used in the field to minimize the risk for infection of workers by rodentborne zoonoses (*17*). Marsupials and rodents were collected in Tomahawk and Sherman traps. Based on data provided by the animal exotic disease division of the Mexican Agricultural Ministry (CPA), the traps were placed in the periphery (usually next to fences) or inside farms that recently reported equine encephalitis. Trapped mammals were collected each morning and transferred to the Agricultural Animal Health Laboratory in Mapastepec, Chiapas, for processing. After being anesthetized with halothane, trapped rodents and marsupials were bled by cardiac puncture, and ≈1 mL of blood was transferred to vacutainers (Becton Dickinson, Franklin Lake, NJ) for serum separation. Some rodents were also bled from the retroorbital sinus with heparinized capillary tubes. Serum specimens were stored in liquid nitrogen for the subsequent analysis at the biosafety level 3 facilities of CPA in Mexico City or at the University of Texas Medical Branch in Galveston, Texas. Dog blood samples were obtained by venipuncture and processed as above. Domestic bird blood samples were collected from the brachial vein. Cattle blood samples were collected from the jugular veins.

### Sentinel Animals

Based on human serosurvey data and to maximize chances for viral isolations, we selected two locations for exposure of sentinel animals: Las Coaches (75% seroprevalence) and Roberto Barrios (54% seroprevalence) (Figure 1). In Las Coaches we placed ten, 6- to 8-week-old Syrian hamsters, 1–2 per cage in hamster-baited mosquito traps as described previously (*18*). The traps were located adjacent to marshes at the eastern part of the village, ≈75 m from human dwellings and close to cattle pastures, swamps, and mangrove forest. In Roberto Barrios, we exposed four sentinel hamsters in open cages, one hamster per cage, at four locations at the western tip of the village, adjacent to marshes and mangrove forest. Hamsters were monitored daily for 7 consecutive days, and brain, spleen, heart, and lung were dissected from moribund (euthanized by halothane overdose) or dead animals and stored in liquid nitrogen.

### Serologic Tests

Initial screening of human serum specimens for VEEV-reactive antibodies was conducted by using 80% plaque reduction neutralization tests (PRNT) with subtype IE strain 68U201, shown previously to be very closely related to VEEV strains circulating on the Pacific coast of Mexico (*19*). Positive samples (>1:20 titer) were further analyzed for immunoglobulin (Ig) M by using an antibody capture enzyme-linked immunoassay (MAC-ELISA) (*20**–**22*). The University of Texas Medical Branch Institutional Review Board approved screening of all human sera.

### Virus Isolation

Heart and brain tissues from sentinel hamsters were triturated in Eagle's minimal essential medium (MEM) containing gentamicin and 20% fetal bovine serum to generate 10% suspensions. After centrifugation at 10,000 x *g* for 5 min, the supernatant was added to Vero cell monolayers and incubated for 4 days or until cytopathic effects were evident. VEEV antigen was identified in the Vero cells by using monoclonal antibodies (*23*), and VEEV RNA was detected in the supernatant by using reverse transcription–polymerase chain reactions (RT-PCR) as described previously (*19*).

### Viral Sequencing and Phylogenetic Analyses

Partial or complete VEEV genomic sequences were determined (*19**,**24*). Viral RNA was extracted from the first BHK cell passage by using Trizol (Gibco BRL, Bethesda, MD), according to the manufacturer's protocol, and subjected to reverse transcription with a primer of sequence T25V designed to anneal to the poly (*25*) tract of the genomic and subgenomic RNA. Complementary DNA was subjected to PCR by using primers to amplify either the complete PE2 envelope glycoprotein precursor gene or the entire viral genome (*24*). PCR amplicons were purified with the Qiagen (Valencia, CA) kit according to the manufacturer's protocol and sequenced directly by using the ABI BigDye kit (Applied Biosystems, Foster City, CA) and an ABI377 automated sequencer. The sequence of the 5´ terminal 20 nucleotides of the genome was not determined because the PCR amplicons incorporated primers corresponding to this region. Sequences were aligned to homologous GenBank sequences for subtype IE VEEV strains subtypes, and other VEE complex sequences were used as outgroups for phylogenetic analyses. Aligned nucleotide and deduced amino acid sequences were analyzed by using the maximum parsimony, neighbor-joining, and maximum likelihood programs in the PAUP 4 package (*26*). Bootstrap values were determined to assess the robustness of topologies with 1,000 replicates (*27*). A relative rates test was used to estimate the rate of evolution for VEEV in Mexico and elsewhere.

### Statistical Methods

Statistical analyses were performed with Stata (Stata corp2001. STATA Statistical software release 7.0. College Station, TX) and EpiInfo 86 (Centers for Disease Control [CDC]-World Health Organization version 6.04 July 1996, CDC, Atlanta, GA). We estimated 95% confidence intervals (CI) and tested risk factors by age, sex, location, and occupation. Where appropriate, chi-square tests and other statistical approaches were used to test differences in risk among populations.

## Results

### Seroprevalence in Wild Animals and Bovines

Animals captured with Tomahawk and Sherman traps were tested for VEEV antibodies by using hemagglutination inhibition (HI), PRNT, or both, depending on the capabilities of the laboratory testing the samples. The results are shown in Table 2. Most notable were opossums, with an overall seropositivity of 25%, cotton rats (*Sigmodon hispidus*) with 67%, and rice rats (*Oryzomys alfaroi*) with 17% seropositivity. Cotton rats were suspected to be the most important reservoir hosts in enzootic VEEV foci studied in other parts of Mexico and Central America (*28*).

**Table 2:** VEEV seroprevalence in wild animals from coastal Chiapas State, Mexico^a^

Species	Locality	No. Collected	Month (2000)	% pos	Titers (test)
*Philander opossum*	Pampa Honda	2	Apr	0	0
(Marsupialia; Didelphidae)	Las Coaches	5	Nov	20	640 (HI)
*Didelphis marsupialis*	La Providencia	1	Jun	100	160 (HI)
(Marsupialia; Didelphidae)					
*Orizomis alfaroi*	Pampa Honda	2	Apr	50	20 (PRNT)
(Rodent; Muridae)	Pampa Honda	4	Aug	0	
*Orizomis couesi* (Rodent; Muridae)	Pampa Honda	2	Apr	0	
	Pampa Honda	1	Aug	0	
	Santa Olga	6	Nov	0	
*Sigmodon hispidus* (Rodent; Muridae)	Pampa Honda	1	Apr	0	
	La Providencia	6	Aug	100	20–160 (HI)
	San Pedro	2	Jun	0	
*Rattus rattus* (Rodent; Muridae)	Pampa Honda	2	Aug	0	
Bovines (1.5 mo age)	Las Coaches	20	Nov	70	20–640 (PRNT)
*Canis familiaris* (dog)	Pampa Honda	3	Jun	33	20 (PRNT)
Chicken	Pampa Honda	5	Jun	20	20 (PRNT)
*Meleagris gallopavo* (turkey)	Pampa Honda	3	Jun	33	20 (PRNT)
*Anser cinereus* (goose)	Pampa Honda	1	Jun	100	320 (PRNT)

^a^VEEV, Venezuelan equine encephalitis virus; HI, hemagglutination inhibition; PRNT, plaque reduction neutralization test; pos, positive.

Simultaneously, we conducted a bovine sentinel serosurvey by testing serum specimens from 20 calves (6–18 months old) from several different farms in and around La Encrucijada. Of these samples, 14 were VEEV-positive by PRNT (titers 20–640); 8 of the calves lived within La Encrucijada Preserve. Subsequently, we bled an additional 110 calves from the same region, and 50 had HI titers from 20–320. Thirty of these also came from La Encrucijada within a radius of ≈15 km of the mouth of the river Novillero. The overall 49% seroprevalence in cattle <18 months of age (lifelong residents of the same ranches, as reported by owners) indicated that bovines are exposed to VEEV in southern Mexico and are excellent sentinels because they are fed on by large numbers of mosquitoes, are susceptible to benign VEEV infection, seroconvert (*29*), and are never vaccinated against VEEV.

### Human Seroprevalence

**Figure 2:**
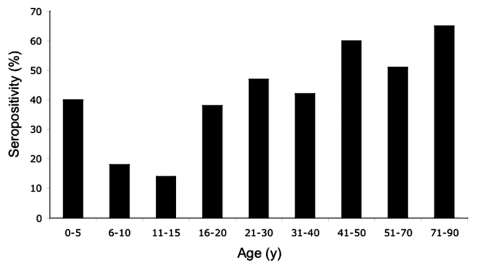
Rates of Venezuelan equine encephalitis virus seropositivity by age group for persons living in the La Encrucijada region. Positive samples had 80% plaque reduction neutralization titers of *1:20. The total numbers of serum specimens tested for each age group were as follows: 0–5 years, 10; 6–10 years, 75; 11–15 years, 89; 16–20 years, 35; 21–30 years, 64; 31–40 years, 76; 41–50 years, 54; 51–70 years, 63; 71–90 years, 14.

Serum samples from 434 persons who resided in La Encrucijada were tested for VEEV antibodies. The PRNT results are summarized by age cohort in Figure 2. The overall PRNT seropositivity in the region was 37.6%; 38.5% of women and 36.4% of men. Among the different communities sampled, seropositivity ranged from 4% (Jamaica) to 75% (Las Coaches; see Table 3). Most notable was the distribution of seropositivity by age, with rising rates in the older age groups (Figure 2). The only exceptions to this trend were in age groups with small sample sizes (e.g., 0–5 years of age, 4/10 positive). Assuming that infection leads to PRNT antibodies lasting >25 years (e.g., human antibody responses to the TC-83 live-attenuated VEEV vaccine can last >30 years (R.E. Shope, pers. comm.), these data are consistent with a steady rate of endemic exposure to VEEV during the past 70 years; there is no suggestion of a disproportionately higher rate in persons >30 years of age, which would be expected from exposure during the 1969–1971 Mexican VEE epizootic. These data indicate long-term endemic circulation of VEEV in the La Encrucijada region.

**Table 3:** VEEV human seroprevalence in 9 localities of Coastal Chiapas sampled from October to December, 2000

Location	No. inhabitants	No. sampled	No. (%) positive	Estimated no. persons exposed
Las Coaches, Pijijiapan	362	36	27 (75)	221
Isla Morelos, Mapastepec	391	43	25 (58)	226
Roberto Barrios, Mapastepec	372	69	37 (54)	201
10 de Abril, Mapastepec	81	16	5 (31)	25
Francisco Sarabia, Mapastepec	354	76	14 (18)	67
Buena Vista, Pijijiapan	440	83	52 (63)	277
Las Palmas, Acapetahua	832	44	8 (18)	151
Jamaica, Escuintla^b^	703	26	1 (4)	28
Cintalapa^b^	632	41	3 (7)	47
Total	4,167	434	172 (40)	1,666

^a^VEEV, Venezuelan equine encephalitis virus. ^b^Cintalapa and Jamaica were the negative controls for our "unexposed" VEEV zone.

Of the PRNT positive human serum specimens, 8 (2%) were positive by IgM capture ELISA, which indicates VEEV infection during the past several months. As was the case for overall seropositivity, the IgM-positive samples were relatively evenly distributed among age groups and sexes (Table 4), which suggests that infections were recent. The low PRNT titers of some of the IgM-positive samples could have reflected differences between antigens on the circulating VEEV strains and those used in the neutralization test, or the titers could have decreased between the time of acute infection.

**Table 4:** Titers of IgM, IgG and PRNT in humans positive for VEEV IgM during the sampling period, October–December, 2000^a^

Community	Sex	Age	IgM titer (ELISA)	IgG titer (ELISA)^b^	PRNT titer
Roberto Barrios	Female	55	100	400	40
10 de Abril	Female	17	1,600	<100	80
10 de Abril	Male	14	400	100	20
Isla Morelos	Female	25	400	400	80
Buena Vista	Female	43	6,400	100	20
Buena Vista	Male	37	400	100	40
Buena Vista	Male	29	100	400	160
Buena Vista	Female	66	100	400	160

^a^Ig, immunoglobulin; PRNT, plaque reduction neutralization tests; VEEV, Venezuelan equine encephalitis virus; ELISA, enzyme-linked immunosorbent assay. ^b^ELISA titers were determined by using fourfold serum dilutions; others were determined by using twofold dilutions.

### Risk Analysis for Human Infection

The risk factor analysis conducted on the inhabitants of the nine sampled villages showed varying results, according to different characteristics of the inhabitants and the geographic location of their households. Statistically significant associations for past VEEV exposure (seropositivity) were linked to occupation, age, and geographic location of the village. Analysis of occupations showed that medical personal had the highest risk (odds ratio [OR] 95.75, 95% CI 10.23–106.93), followed by fisherman (OR 14.39, 95% CI 4.20–52.22), housewives (OR 5.79, 95% CI 2.20–16.10), and farmers (OR 5.10, 95% CI 1.78–15.32). Results for these categories were compared to the results for the high school student population. Additional occupations not statistically associated with VEEV exposure included junior high school and primary students and preschool children. With regard to age, the groups at higher risk of past VEEV exposure were persons 71–90 years of age (OR 8.44, 95% CI 2.18–34.19), followed by persons 51–70 years (OR 4.69, 95% CI 2.13–10.41), 41–50 years (OR 7.37, 95% CI 3.2–17.18), 31–40 years (OR 3,41, 95% CI 1.59–7.36), and 21–30 years (OR 4,54, 95 % CI 2.06–10.11). We compared all the above categories to the age group of children 11–15 years of age. The youngest persons (<10 years of age) had the lowest risk for past exposure, which is consistent with endemic VEEV circulation.

The geographic location or address of people also had a significant association with past VEEV infection, including the physical location of the village with respect to the mangrove forests and marshes. The closer the household was to these habitats, the higher the risk for their inhabitants being seropositive (OR 11.8, 95 % CI 4.03–38.85).

### Isolation of VEEV Strains

Of a total of 14 hamsters exposed in La Encrucijada during July, 2001 (98 hamster-days), 10 became ill or died. Deaths began on the third day after hamster exposure, and VEEV was isolated from the hearts of five animals (Table 5). These viruses were confirmed antigenically as VEEV subtype ID/E by using enzootic subtype-specific monoclonal antibodies and immunofluorescence (*23*). Additionally, several isolates of Group C arboviruses (*Bunyaviridae*: *Orthobunyavirus*) were made.

**Table 5:** Results of Sentinel hamster exposure in La Encrucijada Preserve, July, 2001^a^

Site	No. hamsters exposed	Hamster-days of exposure	No. moribund or dead hamsters	VEEV isolations
Las Coaches	10	70	7	5
Roberto Barrios	4	28	0	0

^a^VEEV, Venezuela equine encephalitis virus.

**Figure 3:**
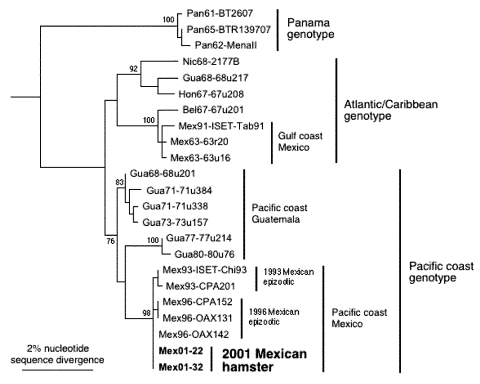
Maximum parsimony phylogenetic tree derived from partial PE2 envelope glycoprotein precursor gene sequences showing relationships of the newly isolated Venezuelan equine encephalitis virus (VEEV) strains from sentinel hamsters (Mex01-22 and Mex01-32) to other subtype IE strains sequenced previously (*19*). Strains are designated by country abbreviation followed by year of isolation and strain designation. Numbers indicate nucleotide substitutions assigned to each branch.

**Figure 4:**
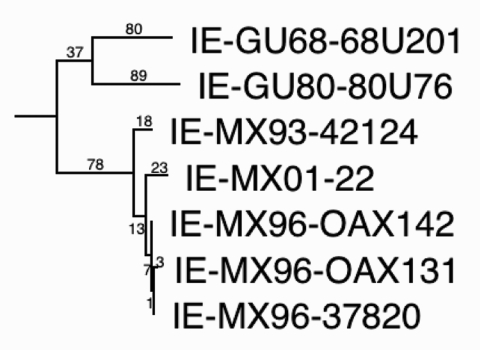
Maximum parsimony phylogenetic tree derived from complete genomic sequences showing relationships of the newly isolated Venezuelan equine encephalitis virus (VEEV) strain (MX01-22) to other strains from Mexico and Guatemala. Numbers indicate nucleotide substitutions assigned to each branch. All nodes had bootstrap values of 100%, except the OAX131-37820 (62%) and GU68-GU80 (<50%) groupings. Relative rates tests applied to the branches indicated a rate of nucleotide substitution in Mexico of 2.0–2.9 x 10–4 subst/nt/y since 1993, and 6.8 x 10–5 for the Guatemalan lineage from 1968–1980. These data suggest continuous circulation of VEEV in Mexico since 1993.

Two of the VEEV strains (MX01-22, MX01-32) from two locations in Las Coaches were selected to assess markers of the epizootic phenotype and genetic relationships to Mexican VEEV isolates from the 1993 and 1996 outbreaks. The partial (856 nucleotides) PE2 envelope glycoprotein precursor gene was sequenced from both isolates (sequences submitted to GenBank), and both sequences were identical and most closely related to subtype IE VEEV isolated from the 1996 Oaxaca outbreak. As in previous analyses (*19*), the Mexican and Pacific Coastal Guatemalan subtype IE isolates grouped independently of the Atlantic/Caribbean Central American and Mexican isolates, as well as the Panama genotype (Figure 3). However, relationships among the Mexican strains from the Pacific coast were not well resolved. Therefore, the complete genome of one strain, MX01-22, was sequenced (submitted to GenBank). The MX01-22 sequence had only 29 (0.26%) different nucleotides and three different deduced amino acids when compared to its closest relative, the 1996 equine strain OAX142; phylogenetic reconstruction showed that the MX01-22 strain is closely related to all of the 1996 equine isolates (Figure 4). Relative rate analyses indicated sequence divergence of 2–2.9 x 10^–4^ subst/nt/year for this Mexican lineage, similar to estimates of other enzootic VEEV lineages in South America (*30*). These data are consistent with the continuous circulation of a single major lineage of subtype IE VEEV in coastal Chiapas and Oaxaca states since 1993. The Guatemalan enzootic lineage represented by the 1968 and 1980 isolates from La Avellana on the Pacific coast had a slightly lower estimated sequence divergence rate of 6.8 x 10^–5^ subst/nt/year.

## Discussion

### Endemic and Enzootic VEE in Coastal Chiapas State

Using a combination of human serosurveys, wild and domestic animal serosurveys, and detection of virus circulation with sentinel hamsters, we obtained evidence that VEEV circulated in the La Encrucijada area of coastal Chiapas State for at least several decades before the 1993 epizootic and continues to circulate in an undescribed transmission cycle. Satellite imagery (Figure 1) and visual inspections indicate that lowland tropical forest habitats characteristic of VEEV enzootic foci, including of subtype IE viruses in other areas of Mexico and the nearby Pacific coast of Guatemala (*31**,**32*), have been almost completely destroyed for the purposes of cattle ranching and other agricultural activities. Mosquito collections in La Encrucijada have indicated an extremely low abundance of *Culex* (*Melanoconion*) *taeniopus*, the proven enzootic vector in a coastal Guatemalan subtype IE VEEV focus (*33*). This indicates that VEEV is likely using a different mosquito species as its enzootic vector in coastal Chiapas State. Experimental studies indicate that adaptation of the Mexican VEEV strains for efficient infection of *Ochlerotatus taeniorhynchus* mosquitoes, through a mutation in the E2 envelope glycoprotein, may have contributed to epizootic transmission (*34*).

Seroprevalence in wild animals suggests a possible role for cotton rats (*S. hispidus*), implicated previously as a VEEV reservoir host in other parts of Mexico (*28*) and in Panama (*35**,**36*). Although the number of animals tested was small, our data also suggest a possible role for rice rats (*Oryzomys alfaroi*) and opossums (*Didelphis marsupialis* and *Philander opposum*) as vertebrate reservoir hosts required to maintain horizontal transmission or amplification hosts involved in increased circulation, resulting in equine cases. Other domestic animals, including dogs, cattle, and birds, are also exposed to VEEV in Chiapas State. Previous experimental studies of dogs (*37*) and cattle (*38*) indicate that low levels of viremia or none develops in those animals after VEEV infection and, therefore, they are probably not important as enzootic hosts. Larger sample sizes and experimental infections to assess viremia levels are needed before conclusions can be drawn regarding the relative importance of different animals as reservoir or amplification hosts of VEEV in Mexico.

The vertebrate amplification hosts responsible for increased circulation of VEEV and its transmission to equines during 1993 and 1996 remain unidentified, since equines inefficiently amplify the etiologic VEEV strains of the subtype isolated during the outbreaks (*16*). One possibility for an alternative amplification host is humans, in whom high levels of viremia develop after infection by subtypes IAB and IC VEEV (*14**,**39*). Our results indicate that persons are regularly exposed in coastal Chiapas, and their migratory work habits could enhance spread during outbreaks. Estimation of viremia titers from infected persons in southern Mexico is needed to test this hypothesis. Another possibility is that increased populations of wild vertebrate hosts such as *S. hispidus* resulted in increased amplification of the virus and its transmission to equines during the summer of 1993.

### Human Risk and Disease

Although our data indicate that persons are regularly infected with VEEV in coastal Chiapas State, the effect of endemic transmission on human health is unknown. Most undifferentiated febrile illness in the region is clinically diagnosed as dengue or flulike febrile illness, and currently no diagnostic tools are in place to test for human VEEV infections. We identified the occupations of La Encrucijada inhabitants and the ecologic habitats where they live and work as risk factors for VEEV infection. We detected no risk differences between the sexes. A possible explanation is that, in this region, female homemakers often accompany male farm laborers to sites where VEEV may circulate.

The spatial risks for VEEV infection apparently do not include proximity to human habitations. Thus, VEEV is distinct from dengue viruses, which circulate peridomestically (*40*). The higher risk for medical personnel for VEEV infection suggests the possibility of aerosol exposure in clinical settings. Epidemiologic data on the seasonality of flulike febrile illness in coastal Chiapas State (Instituto Mexicano del Seguro Social, unpub. data) indicate peak incidence from June to November, coincident with the rainy season and peak mosquito populations. These data suggest that many flulike illnesses may be caused by VEEV and possibly other arboviruses such as group C orthobunyaviruses.

## References

[1] Beck CE, Wyckoff RWG. Venezuelan equine encephalomyelitis. Science. 1938;88:530. 10.1126/science.88.2292.530 17840536

[2] Albornoz JE. La peste loca de las bestias (Enfermedad de Borna). Colombia. Min Agr Com, Bogota. Bol de Agr (Suppl). 1935;26:1–8.

[3] de Mucha-Macias J. Infecciones por virus arbor. Gac Med Mex. 1963;93:415–20. 14025971

[4] de Mucha-Macias J, Sanchez-Spindola I, Campillo-Sainz C. Venezuelan equine encephalomyelitis antibodies in human beings of southeastern Mexico. Am J Trop Med Hyg. 1966;15:364–8. 5949344 10.4269/ajtmh.1966.15.364

[5] Zarate ML, Scherer WF, Dickerman RW. El virus de la encephalitis equina de Venezuela como determinante de infecciones en humanos, descripción de un caso fatal ocurrido en Jaltipán Veracruz en 1965. Rev Invest Salud Publica. 1965;30:296–302. 5516706

[6] de Mucha-Macias J. Encefalitis equina de Venezuela en Tamaulipas, Mexico. Rev Invest Salud Publica (Mexico). 1966;26:277–9. 5993039

[7] Scherer WF, Dickerman RW, Chia CW, Ventura A, Moorhouse A, Geiger R, Venezuelan equine encephalitis virus in Veracruz, Mexico, and the use of hamsters as sentinels. Science. 1963;145:274–5. 10.1126/science.145.3629.274 14171568

[8] Morilla-Gonzales A, de Mucha-Macias J. Estudio de una epizootia de encefalitis equina de Venezuela ocurrida en Tamaulipas, Mexico. Rev Invest Salud Publica (Mexico). 1969;29:3–20. 5388000

[9] Sudia WD, Newhouse VF, Beadle ID, Miller DL, Johnston JG Jr, Young R, Epidemic Venezuelan equine encephalitis in North America in 1971: vector studies. Am J Epidemiol. 1975;101:17–35. 235212 10.1093/oxfordjournals.aje.a112068

[10] Batalla-Campero D. Adaptación, modificaciones y pruebas de campo para el desarrollo de una vacuna contra la Encefalitis Equina Venezolana (EEV). In: Trabajos deingreso de Académicos Numerarios y Correspondientes. Mexico City: Academia Veterinaria Mexicana AC; 1995. p. 16–24.

[11] Calisher CH. Medically important arboviruses of the United States and Canada. Clin Microbiol Rev. 1994;7:89–116. 8118792 10.1128/cmr.7.1.89 PMC358307

[12] Walton TE, Grayson MA. Venezuelan equine encephalomyelitis. In: Monath TP. The arboviruses: epidemiology and ecology, vol. IV. Boca Raton (FL): CRC Press; 1988. p. 203–31.

[13] Rico-Hesse R, Weaver SC, de Siger J, Medina G, Salas RA. Emergence of a new epidemic/epizootic Venezuelan equine encephalitis virus in South America. Proc Natl Acad Sci U S A. 1995;92:5278–81. 10.1073/pnas.92.12.5278 7777497 PMC41677

[14] Weaver SC, Salas R, Rico-Hesse R, Ludwig GV, Oberste MS, Boshell J, Re-emergence of epidemic Venezuelan equine encephalomyelitis in South America. VEE Study Group. Lancet. 1996;348:436–40. 10.1016/S0140-6736(96)02275-1 8709783

[15] Oberste MS, Fraire M, Navarro R, Zepeda C, Zarate ML, Ludwig GV, Association of Venezuelan equine encephalitis virus subtype IE with two equine epizootics in Mexico. Am J Trop Med Hyg. 1998;59:100–7. 9684636 10.4269/ajtmh.1998.59.100

[16] Gonzalez-Salazar D, Estrada-Franco JG, Carrara AS, Aronson JF, Weaver SC. Equine Amplification and virulence of subtype IE Venezuelan equine encephalitis viruses isolated during the 1993 and 1996 Mexican epizootics. Emerg Infect Dis. 2003;9:161–8. 12603985 10.3201/eid0902.020124 PMC2901937

[17] Mills JN, Childs JE, Ksiazek TG, Peters CJ. Methods for trapping and sampling small mammals for virologic testing. Atlanta: U.S. Department of Health and Human Services; 1995. p. 61.

[18] Ferro C, Boshell J, Moncayo AC, Gonzalez M, Ahumada ML, Kang W, Natural enzootic vectors of Venezuelan equine encephalitis virus, Magdalena Valley, Colombia. Emerg Infect Dis. 2003;9:49–54. 12533281 10.3201/eid0901.020136 PMC2873762

[19] Oberste MS, Schmura SM, Weaver SC, Smith JF. Geographic distribution of Venezuelan equine encephalitis virus subtype IE genotypes in Central America and Mexico. Am J Trop Med Hyg. 1999;60:630–4. 10348239 10.4269/ajtmh.1999.60.630

[20] Chu YK, Rossi C, Leduc JW, Lee HW, Schmaljohn CS, Dalrymple JM. Serological relationships among viruses in the *Hantavirus* genus, family *Bunyaviridae.* Virology. 1994;198:196–204. 10.1006/viro.1994.1022 8259655

[21] Schmaljohn C, Vanderzanden L, Bray M, Custer D, Meyer B, Li D, Naked DNA vaccines expressing the prM and E genes of Russian spring summer encephalitis virus and Central European encephalitis virus protect mice from homologous and heterologous challenge. J Virol. 1997;71:9563–9. 9371620 10.1128/jvi.71.12.9563-9569.1997 PMC230264

[22] Tardei G, Ruta S, Chitu V, Rossi C, Tsai TF, Cernescu C. Evaluation of immunoglobulin M (IgM) and IgG enzyme immunoassays in serologic diagnosis of West Nile virus infection. J Clin Microbiol. 2000;38:2232–9. 10834982 10.1128/jcm.38.6.2232-2239.2000 PMC86770

[23] Roehrig JT, Bolin RA. Monoclonal antibodies capable of distinguishing epizootic from enzootic varieties of Subtype I Venezuelan equine encephalitis viruses in a rapid indirect immunofluorescence assay. J Clin Microbiol. 1997;35:1887–90. 9196217 10.1128/jcm.35.7.1887-1890.1997 PMC229865

[24] Brault AC, Powers AM, Holmes EC, Woelk CH, Weaver SC. Positively charged amino acid substitutions in the E2 envelope glycoprotein are associated with the emergence of Venezuelan equine encephalitis virus. J Virol. 2002;76:1718–30. 10.1128/JVI.76.4.1718-1730.2002 11799167 PMC135911

[25] Encephalitis, Venezuelan equine, 30 years of study in Venezuela, 1963–1993. Invest Clin. 1995;36(Suppl 2):1–565. 8911032

[26] Swofford DL. PAUP*. Phylogenetic Analysis Using Parsimony (*and Other Methods). Version 4. Sunderland (MA): Sinauer Associates; 1998.

[27] Felsenstein J. Confidence limits on phylogenies: an approach using the bootstrap. Evolution. 1985;39:783–91. 10.2307/2408678 28561359

[28] Scherer WF, Dickerman RW, La Fiandra RP, Wong Chia C, Terrian J. Ecologic studies of Venezuelan encephalitis virus in southeastern Mexico. IV. Infections of wild mammals. Am J Trop Med Hyg. 1971;20:980–8. 5131697 10.4269/ajtmh.1971.20.980

[29] Walton TE, Johnson KM. Experimental Venezuelan equine encephalomyelitis virus infection of the bovine. Infect Immun. 1972;5:155–9. 4564396 10.1128/iai.5.2.155-159.1972 PMC422339

[30] Brault AC, Powers AM, Medina G, Wang E, Kang W, Salas RA, Potential sources of the 1995 Venezuelan equine encephalitis subtype IC epidemic. J Virol. 2001;75:5823–32. 10.1128/JVI.75.13.5823-5832.2001 11390583 PMC114297

[31] Dickerman RW, Scherer WF, Diaz-Najera A. Ecologic studies of Venezuelan encephalitis virus in southeastern Mexico. I. Introduction and study sites. Am J Trop Med Hyg. 1971;20:730–9. 5093671 10.4269/ajtmh.1971.20.730

[32] Scherer WF, Dickerman RW, Ordonez JV, Seymour C III, Kramer LD, Jahrling PB, Ecologic studies of Venezuelan encephalitis virus and isolations of Nepuyo and Patois viruses during 1968–1973 at a marsh habitat near the epicenter of the 1969 outbreak in Guatemala. Am J Trop Med Hyg. 1976;25:151–62. 3981 10.4269/ajtmh.1976.25.151

[33] Cupp EW, Scherer WF, Ordonez JV. Transmission of Venezuelan encephalitis virus by naturally infected *Culex* (*Melanoconion*) *opisthopus.* Am J Trop Med Hyg. 1979;28:1060–3. 507283 10.4269/ajtmh.1979.28.1060

[34] Brault AC, Powers AM, Ortiz D, Estrada-Franco JG, Navarro-Lopez R, Weaver SC. Venezuelan equine encephalitis emergence: enhanced vector infection from a single amino acid substitution in the envelope glycoprotein. Proc Natl Acad Sci U S A. 2004;101:11344–9. 10.1073/pnas.0402905101 15277679 PMC509205

[35] Grayson MA, Galindo P. Ecology of Venezuelan equine encephalitis virus in Panama. J Am Vet Med Assoc. 1969;155:2141–5. 4904962

[36] Young NA, Johnson KM, Gauld LW. Viruses of the Venezuelan equine encephalomyelitis complex. Experimental infection of Panamanian rodents. Am J Trop Med Hyg. 1969;18:290–6. 5777742

[37] Dickerman RW, Scherer WF, Navarro E, Ordonez M, Ordonez JV. The involvement of dogs in endemic cycles of Venezuelan encephalitis virus. Am J Epidemiol. 1973;98:311–4. 4743343 10.1093/oxfordjournals.aje.a121560

[38] Dickerman RW, Baker GJ, Ordonez JV, Scherer WF. Venezuelan equine encephalomyelitis viremia and antibody responses of pigs and cattle. Am J Vet Res. 1973;34:357–61. 4691483

[39] Bowen GS, Calisher CH. Virological and serological studies of Venezuelan equine encephalomyelitis in humans. J Clin Microbiol. 1976;4:22–7. 956360 10.1128/jcm.4.1.22-27.1976 PMC274383

[40] Gubler DJ. Dengue and dengue hemorrhagic fever: its history and resurgence as a global public health problem. In: Gubler DJ, Kuno G. Dengue and dengue hemorrhagic fever. New York: CAB International; 1997. p. 1–22.

